# Long Noncoding RNAs Act as Novel Biomarkers for Hepatocellular Carcinoma: Progress and Prospects

**DOI:** 10.1155/2017/6049480

**Published:** 2017-08-01

**Authors:** Han Bao, Hongying Su

**Affiliations:** Department of Radiology, The First Hospital of China Medical University, 155 Nanjing North Street, Shenyang, Liaoning 110001, China

## Abstract

Hepatocellular carcinoma (HCC) is one of the most common cancers worldwide and confers a poor prognosis. Novel diagnostic or prognostic biomarkers and effective therapeutic targets for HCCs are urgently needed. Currently, dozens of long noncoding RNAs (lncRNAs) have been identified as playing critical roles in cancer development and progression. Advanced studies have shown that several well-known lncRNAs are dysregulated in HCC tissue as compared to adjacent noncancerous tissue. Furthermore, highly stable cell-free circulating nucleic acids (cfCNAs), including lncRNAs, aberrantly expressed in the plasma of HCC patients, have been detected. In this review, we focus on the most extensively investigated lncRNAs in HCC and discuss the potential of HCC-related lncRNAs as novel biomarkers for early diagnosis and prognosis.

## 1. Introduction

Hepatocellular carcinoma (HCC) is the most common liver cancer worldwide and has a high morbidity rate in Eastern Asia and several African countries [1]. The high mortality rate and poor prognosis in HCC patients are largely associated with lack of apparent symptoms, frequent tumor metastasis, and ineffective therapeutic options [2]. Therefore, a better understanding of the molecular mechanisms involved in HCC is needed to discover novel diagnostic and prognostic biomarkers and effective therapeutic targets.

Many studies on the molecular mechanisms involved in hepatocarcinogenesis in recent decades have focused primarily on oncogenic or anticancer protein coding genes. DNA tiling array technology and deep sequencing have led to more studies on noncoding RNAs (ncRNAs), including short (<200 nucleotides) and long transcripts (>200 nucleotides). Long noncoding RNAs (lncRNAs) were previously regarded as transcriptional background noise [3–5]. However, recent studies have shown that lncRNAs transcribed by RNA polymerase II play critical roles in regulating many different biological processes involved in HCC progression, including angiogenesis, cell proliferation, apoptosis, invasion, and metastasis [6]. Several well-known lncRNAs have been shown to be dysregulated in HCC tissues compared to adjacent noncancerous tissues [7]. Several HCC-related lncRNAs have been suggested as novel biomarkers for diagnosing HCC and predicting prognosis and response to therapy due to their presence in the plasma, good specificity, and accessibility [8].

In this review, we focus on HCC-related lncRNAs with aberrant expression in tumor tissues and their mechanisms. Furthermore, we summarize lncRNAs that are dysregulated in the plasma of HCC patients and their diagnostic value as novel biomarkers. Although few studies on lncRNAs in HCC have been published, understanding the unique roles played by lncRNAs in HCC progression will be important in therapeutic decision-making.

## 2. Dysregulated Long Noncoding RNAs in HCC Tissues

Several HCC-related lncRNAs have been demonstrated to play irreplaceable roles in the progression of hepatocellular carcinoma (see Table 1). Although the underlying mechanism of HCC-related lncRNAs remains unknown, the crucial biological functions of lncRNAs are often associated with certain signaling pathways, accompanying obvious expression of the disorder in liver cancer tissues. These dysregulated lncRNAs are expected to become novel biomarkers for diagnosis or the evaluation of therapeutic efficiency. Here we highlight five comparatively known HCC-related lncRNAs:* HULC, MALAT1, HOTAIR, MVIH, *and* PVT1*.

### 2.1. *HULC*

Highly upregulated in liver cancer* (HULC)* is located on chromosome 6p24.3 and is approximately 500 nt in size. Panzitt et al. first indicated that* HULC* is specifically expressed in hepatocytes and highly upregulated in HCC [9]. Wang et al. provided evidence that upregulated HULC played an important role in tumorigenesis through inhibiting miR-372 [10]. Du et al. reported that* HULC* expression was positively correlated with hepatitis B virus X protein (HBx) expression in HCC tissues. Further research showed that HBx activates the* HULC *promoter via the CREB and that* HULC* upregulation enhances hepatoma cell proliferation by suppressing p18 [11]. Additionally, Cui et al. hypothesized that* HULC* may affect malignancy by causing lipid metabolism in hepatoma cells. They reported that* HULC* is an oncogene that alters lipid metabolism through a signaling pathway involving miR-9, PPARA, and ACSL1 [12]. Another report from Cui et al. showed that* HULC* influences hepatocarcinogenesis by changing circadian rhythms through circadian oscillator clock circadian regulator (CLOCK) upregulation in hepatoma cells [13]. In addition, Lu et al. reported a positive correlation between* HULC* levels and sphingosine kinase 1 (SPHK1) levels, along with levels of the byproduct sphingosine-1-phosphate (S1P) in HCC tissues. The authors concluded that* HULC* promotes tumor angiogenesis in liver cancer via miR-107/E2F1/SPHK1 signaling [14]. Recently, Li et al. found that HULC was aberrantly upregulated in HCC tissues and associated with TNM stage, intrahepatic metastases, HCC recurrence, and postoperative survival. Further research showed that* HULC* can promote tumor invasion and metastasis of HCC by ZEB1-induced epithelial-mesenchymal transition [15].

### 2.2. *MALAT1*

*MALAT1* (metastasis-associated lung adenocarcinoma transcript 1) is a nuclear lncRNA that is over 8000 nt and originates from chromosome 11q13.* MALAT1* is highly conserved across several species, which indicates functional importance [16]. Lin et al. identified lncRNA transcript Hepcarin (HCN), most likely a murine ortholog of* MALAT1*, which is a novel marker for HCC induced by carcinogens and is highly expressed in murine colon carcinomas. The authors indicated that* MALAT1* has a high sensitivity for human HCCs and suggested it as a potential diagnostic technique [16]. The role of* MALAT1* in HCC prognosis was first examined by Lai et al. The authors reported that* MALAT1* levels were increased in tissue samples from HCC patients and cell lines and showed that shorter disease-free survival was linked with higher* MALAT1* expression levels in HCC patients who had undergone a liver transplant (LT).* MALAT1* expression was shown to be an independent prognostic factor for HCC recurrence after LT, aside from tumor size and PVTT (portal vein tumor thrombus).* MALAT1* expression had prognostic significance in advanced HCC patients and patients with larger tumors (diameters over 5 cm). This information can be used to prescreen HCC patients at more advanced stages prior to LT. In addition to prognostic characteristics,* MALAT1* may also be considered as a therapeutic target given that* MALAT1* inhibition in HepG2 cells was shown to increase sensitivity to apoptosis and reduce cell viability and invasive properties [17]. Luo et al. recently demonstrated overexpression of* MALAT1* in HCCs and human sera collected following arsenite exposure. Hypoxia-inducible factor- (HIF-) 2*α* was also shown to be upregulated in HCCs.* MALAT1* expression induced by arsenite leads to disassociation of the von Hippel-Lindau (VHL) protein from HIF-2*α* and reduces VHL-mediated HIF-2*α* ubiquitination, causing HIF-2*α* to accumulate. As a result, HIF-2*α* transcriptionally regulates* MALAT1*, resulting in a MALAT1/HIF-2*α* feedback loop that influences arsenite-related carcinogenesis [18]. SIRT1 (silent information regulator 1) is a conserved NAD+-dependent histone deacetylase that has been associated with HCC, alcoholic liver disease, and nonalcoholic fatty liver. Wu et al. reported that LX-2 cell activation induced by TGF-*β*1 is affected by SIRT1 overexpression, which may drive cells back to a quiescent state and could be a potential target for treating liver fibrosis. In addition,* MALAT1* may mediate SIRT1 expression and function in cases of liver fibrosis [19].

### 2.3. *HOTAIR*

The lncRNA HOX transcript antisense RNA* (HOTAIR)* was originally discovered by Rinn et al. on chromosome 12q13.13 [20].* HOTAIR* targets the PRC2 complex to the HOXD locus and regulates gene expression by delocalizing PRC2, which is a histone H3 lysine 27 methylase associated with gene silencing and cancer progression, and trimethylation of H3K27 [21]. Expression, clinical significance, and biological roles of* HOTAIR* in HCC tumor progression were first evaluated by Yang et al. The authors reported that* HOTAIR* expression levels in cancer tissues were higher than in adjacent control tissues. High* HOTAIR* expression levels in LT patients were associated with significantly shorter recurrence-free survival (RFS) [22]. Geng et al. reported that patients who had tumors with high* HOTAIR* gene expression were at greater risk of recurrence following hepatectomy.* HOTAIR* expression and lymph node metastasis were also shown to be correlated. Levels of matrix metallopeptidase-9 (MMP-9) and vascular endothelial growth factor (VEGF), which are important for cell motility and metastasis, were shown to be reduced following* HOTAIR* knockdown [23]. Ishibashi et al. showed that* HOTAIR* expression was related to significantly poorer prognoses and larger primary tumors and that* HOTAIR* introduced to liver cancer cells resulted in rapid proliferation compared to controls [24], while Ding et al. reported that* HOTAIR* promoted HCC cell migration and invasion by inhibiting RBM38 (RNA binding motif protein 38) [25]. Fu et al. reported that tumorigenesis was suppressed in HCC after silencing* HOTAIR*, which activated P16 and P14 signaling via increased and decreased miR-218 and Bmi-1 expression, respectively [26]. Gao et al.'s study indicated that* HOTAIR* expression was associated with poor tumor differentiation, metastasis, and early recurrence due in part to effects on the Wnt/*β*-catenin signaling pathway [27]. Yang et al. recently reported that* HOTAIR* activated autophagy through increased autophagy-related 3 (ATG3) and ATG7 expression, thereby enhancing HCC cell proliferation [28].

### 2.4. *MVIH*

Yuan et al. first identified the lncRNA* MVIH* (lncRNA associated with microvascular invasion in HCC) and showed significant upregulation in HCC.* MVIH* overexpression was associated with increased microvascular invasion, a higher tumor node metastasis (TNM) stage, and decreased overall survival (OS) and RFS in a cohort of 215 HCC patients.* MVIH* upregulation was shown to be an independent risk factor in predicting poor RFS, and subsequent research indicated that* MVIH* may activate angiogenesis by inhibiting phosphoglycerate kinase 1 (PGK1) secretion.* MVIH* expression was shown to be inversely correlated with PGK1 serum levels and positively correlated with microvessel density in 65 patients with HCC [29]. Shi et al. reported that* MVIH* likely plays a biological role in HCC tumorigenesis after reporting a significant increase in* MVIH* expression and a decrease in miR-199a expression in tumor tissues and HCC cells. Although si-MVIH reduced HCC cell viability and increased cell apoptosis, an miR-199a inhibitor reversed these effects, indicating that* MVIH* can inhibit apoptosis and increase HCC cell growth by inhibiting miR-199a expression [30].

### 2.5. *PVT1*

*PVT1* is a large (>300 kb) locus located downstream of c-myc on chromosome 8q24. It produces a variety of spliced noncoding RNAs and six annotated microRNAs as follows: miR-1204, miR-1205, miR-1206, miR-1207-5p, miR-1207-3p, and miR-1208. Barsotti et al. identified* PVT1* as a p53-inducible target gene [31]. In addition to reporting that human* lncRNA-hPVT1* was upregulated in HCC tissues, Wang et al. found that higher* lncRNA-hPVT1 *expression was associated with a poor clinical prognosis. The authors confirmed that regulating the lncRNA-hPVT1/NOP2 pathway may be a therapeutic target in treating HCC given that* lncRNA-hPVT1* was shown to promote cell proliferation, cell cycling, and stem cell-like properties in HCC cells by stabilizing the NOP2 protein [32]. Additionally, Ding et al. determined* PVT1* expression in HCCs by RT-qPCR in two independent cohorts and found that relative* PVT1* expression levels were significantly higher in cancerous tissues. Kaplan-Meier and multivariate analyses indicate that patients with high* PVT1* expression have an unfavorable RFS and are independent predictors of HCC recurrence [33].

## 3. Dysregulated lncRNAs in the Sera of HCC Patients

Wide variations in lncRNA expression levels have been described in tumor tissues compared to normal tissues [34]. However, clinical biomarkers should be easily accessible and require noninvasive detection and sampling. Nowadays, an increasing number of circulating lncRNAs have been demonstrated to be dysregulated in plasma or serum, which demonstrates their high potential as powerful and noninvasive tumor markers. [8] (see Table 2). Here, we summarized several lncRNAs which had been reported to be dysregulated in the plasma of HCC patients.

### 3.1. *HULC* and* Linc00152*

In 2013, Xie et al. first reported that lncRNA* HULC* in plasma could be a promising novel biomarker for diagnosing HCC after it was detected by qRT-PCR at a higher frequency in the plasma of HCC patients (*n* = 30) compared to healthy controls (*n* = 20). Increased* HULC* expression has been positively correlated with Edmondson histological grades and hepatitis B (HBV) positive status in tumor tissues, and, as expected, higher detection rates were also observed in the plasma of patients with higher Edmondson grades or with an HBV+ status. These findings indicate differential expression of* HULC* in the serum and tissues of HCC patients [35].

Another diagnosis study about* HULC* was from Li et al. Of eight possible lncRNA candidates (*UCA1, TUG1, CCAT1, MEG3, Linc00152, HULC*,* MALAT1*, and* GAS5*), circulating* HULC* and* Linc00152* were significantly upregulated in plasma samples from HCC patients during training and validation sets using qRT-PCR, with areas under the ROC curves of 0.78 and 0.85, respectively. Combining* HULC* and* Linc00152* resulted in moderate discrimination between HCC and controls, with an area under the ROC curve of 0.87. Both* HULC* and* Linc00152* serum levels show diagnostic accuracy in HCC diagnosis and may act as novel biomarkers [36].

### 3.2. *uc003wbd* and* AF085935*

Lu et al. determined that serum levels of* lncRNA-uc003wbd* and* lncRNA-AF085935* were significantly higher in HCC and HBV patients compared to controls.* lncRNA-AF085935* was relatively more accurate in screening for HCC (AUC = 0.96) than* lncRNA-uc003wbd* (AUC = 0.86) in healthy controls and HBV patients (AUC = 0.70). Serum* lncRNA-uc003wbd* and* lncRNA-AF085935* levels were both altered in HCC and HBV patients, suggesting that these lncRNAs are potential plasma biomarkers [37].

### 3.3. *RP11-160H22.5*,* XLOC_014172*, and* LOC149086*

Tang et al. discovered three lncRNAs in plasma samples from 217 HCC patients by high throughput microarray detection. They reported that* RP11-160H22.5*,* XLOC_014172*, and* LOC149086* were upregulated in HCC as compared to cancer-free controls, with merged AUC in training and validation set at 0.999 and 0.896, respectively. Furthermore,* XLOC_014172* and* LOC149086* were confirmed to be highly increased in metastatic HCC patients with merged AUC in training and validation set at 0.900 and 0.934, respectively. Most patients presented with decreased levels of all three lncRNAs after surgery, while secondary increased levels might be associated with tumor hematogenous metastasis. In conclusion, Tang et al. identified three lncRNAs,* RP11-160H22.5*,* XLOC_014172*, and* LOC149086*, as potential biomarkers for tumorigenesis prediction, and* XLOC_014172* and* LOC149086* for metastasis prediction [38].

### 3.4. *HEIH*

 Zhefeng et al. collected blood samples from 179 HCC and 179 matched healthy controls for quantitative analysis of plasma* lncRNA-HEIH* using qRT-PCR. Forty plasma samples were collected from these patients 7 days after surgery to examine the change in* lncRNA-HEIH *expression. As a result, HCC patients exhibited statistically higher levels of plasma* lncRNA-HEIH* than matched controls. The expression levels of* HEIH* in plasma from postoperative patients were significantly lower than those from preoperative patients. Individuals who had higher plasma* lncRNA-HEIH* expression had a significantly increased risk of HCC when healthy controls were used as the reference group. In addition, the area under the ROC curve for plasma* lncRNA-HEIH* from all participants was 0.681. The combined model of AFP and* lncRNA-HEIH* shows a significantly better prediction efficacy for HCC than either AFP or* lncRNA-HEIH* alone, with the largest AUC (0.714) [39].

### 3.5. *UCA1* and* WRAP53*

Kamel et al. showed that* lncRNA-UCA1* and* lncRNA-WRAP53* expression in HCC patient sera was significantly higher in HCC patients compared to patients with chronic hepatitis C virus (HCV) and healthy volunteers. Serum and tissue levels of these two genes were strongly correlated, and combining both lncRNAs with serum AFP resulted in 100% sensitivity. Thus, increased levels of* lncRNA-UCA1* and* lncRNA-WRAP53* may be novel serum diagnostic biomarkers for HCC [40].

### 3.6. *MALAT1*

Research conducted by Konishi et al. indicated that plasma* MALAT1* levels and liver damage are related, which suggests clinical utility in predicting HCC. The authors collected plasma samples from preoperative HCC patients (*n* = 88), hepatic disease patients (*n* = 28), and healthy controls (*n* = 51) and measured plasma* MALAT1* using qRT-PCR. The expression levels of MALAT1 were significantly higher in HCC patients than in hepatic disease patients. Patients with hepatic disease had higher levels than healthy controls. HCC patients with liver damage or cirrhosis had significantly elevated* MALAT1* levels. ROC analysis between HCC and hepatic disease patients showed a cut-off value of 1.60, with an AUC of 0.66. Sensitivity and specificity for HCC in combination with plasma* MALAT1*, AFP, and PIVKAII were 88.6% and 75%, respectively, indicating that a combination of factors is more accurate for diagnosing HCC than individual parameters [41].

### 3.7. *uc001ncr* and* AX800134*

Wang et al. collected 684 blood samples from patients with chronic HBV, HBV-positive HCC, and healthy volunteers and separated the samples into three phases. Differential expression of lncRNAs were determined using microarrays, leading to selection of* uc001ncr* and* AX800134* as candidates due to significant upregulation in HCC tissue and serum samples. These two lncRNAs were subsequently analyzed by qRT-PCR in serum samples obtained from an independent cohort of 353 participants (121 HBV-positive HCC patients, 95 HBV patients, and 137 healthy controls). Additional serum samples from 61 HBV-positive HCC patients, 60 HBV patients, and 60 healthy individuals were also used to validate the diagnostic efficacy of the two lncRNAs. Based on* uc001ncr* and* AX800134* expression, these lncRNAs were determined to accurately diagnose HBV-positive HCC (AUC = 0.9494), including patients with an AFP of 400 ng/ml (AUC = 0.9371), indicating potential usefulness as biomarkers in diagnosing HCC [42].

### 3.8. *PVT1* and* uc002mbe.2*

Recently, Yu et al. studied cancer-related lncRNAs in sera obtained from 71 HCC patients and 64 healthy controls using qRT-PCR. lncRNA* PVT1* and* uc002mbe.2* showed significantly upregulated expression levels in HCCs, and the combination of* PVT1* and* uc002mbe.2* was considered a possible HCC marker, with an area under the ROC curve of 0.764 and sensitivity and specificity of 60.56% and 90.62%, respectively. The serum 2-lncRNA signature combined with AFP yielded greater diagnostic value than AFP alone [43].

The above studies have shown that plasma lncRNAs have enormous potential to serve as biomarkers for HCC diagnosis due to their stability, relationship with hepatocellular carcinoma, and easy acquisition. Serum lncRNAs can be screened for early diagnosis, to evaluate surgical or nonsurgical therapeutic efficiency, to monitor recurrence during the follow-up period, and to provide new therapeutic targets.

## 4. Conclusion and Future Perspectives

In conclusion, many studies have suggested that lncRNAs are vital in various biological processes involved in HCC, such as initiation, progression, metastasis, recurrence, treatment and prognosis. Moreover, dysregulation of HCC-related lncRNAs in tumor tissues is generally associated with these biological processes.

HCC is a common cancer worldwide, with a poor prognosis due to difficulties in early discovery, diagnosis, and treatment. Traditional serum markers of HCC, for example AFP, are limited by low sensitivity and specificity [34]. Therefore, accurate novel biomarkers and efficient therapeutic targets for diagnosing and treating HCC are needed. An increasing number of studies have demonstrated that expression of several lncRNAs is dramatically dysregulated, not only in tumor tissues but also in patient plasma samples. Given that cancer-specific miRNAs are detectable in various biological fluids, they are being actively investigated as potential diagnostic and prognostic biomarkers [44]. Similarly, detection of lncRNAs in serum has promising utility as a noninvasive clinical technique.

Existing studies showed that lncRNA could keep stable expression in plasma when sera is treated with a prolonged room temperature incubation, multiple freeze-thaw cycles, a low or high pH solution, or RNase A digestion. Moreover, several researches showed that HCC-related lncRNAs are both dysregulated in tissues and sera. We suspect that lncRNAs which are dysregulated in the plasma of HCC patients were released by tumor tissues and circulating lncRNAs may be protected by extracellular vesicles, including apoptotic bodies, microvesicles, and exosomes. However, there is no direct evidence that proved our point. Another problem is that the results of the different researchers on the same gene may not be consistent due to the population genetic difference.

In summary, existing knowledge surrounding lncRNAs is limited, and the diverse functions lncRNAs may play a role in HCC development in the early stages of discovery. Understanding the molecular mechanisms of HCC-related lncRNAs will be useful in determining potential future molecular targets. Further investigation will be necessary to unravel the regulatory networks and molecular characteristics of lncRNAs in an effort to ultimately streamline and improve HCC diagnosis and treatment.

## Figures and Tables

**Table 1:** Dysregulated long noncoding RNAs (lncRNAs) in HCC tissues.

Name	Dysregulation	Biological functions in HCC	Reference
*HULC*	Up	Promote tumor genesis, metastasis and angiogenesis; support abnormal lipid metabolism	[10–15, 45]

*MALAT1*	Up	Regulate arsenite-induced malignant transformation and alternative splicing of pre-mRNA, promote liver fibrosis and tumor recurrence	[17–19, 46]

*HOTAIR*	Up	Associated with poor tumor differentiation, metastasis and early recurrence, promote migration and invasion, activate autophagy	[22, 23, 25–28]

*HOTTIP*	Up	Associated with metastasis formation and poor survival	[47–49]

*MVIH*	Up	Active angiogenesis, promote tumor growth and intrahepatic metastasis, inhibit cell apoptosis	[29, 30]

*HEIH*	Up	Regulate the cell cycle, promote tumor progression	[50]

*PVT1*	Up	Promote cell proliferation, cell cycle, and stem cell-like properties; associated with tumor suppression	[31, 32]

*CCAT1*	Up	Promote proliferation and migration, function as a molecular sponge	[51]

*URHC*	Up	Promote cell proliferation and inhibit apoptosis	[52]

*GIHCG*	Up	Promote xenografts growth and metastasis	[53]

*Linc00974*	Up	Promote cell proliferation and invasion	[54]

*H19*	Up	Induce drug resistance, promote tumor progression and invasion	[55–57]
	Down	Suppress intrahepatic metastasis, suppress migration and invasion of HCC cells	[58, 59]

*MEG3*	Down	Inhibit tumor cell proliferation	[60–66]

*hDreh*	Down	Inhibit tumor growth and metastasis	[67]

*LET*	Down	Inhibit tumor metastasis under hypoxic conditions	[68]

**Table 2:** Dysregulated lncRNAs in serum of HCC patients.

Name	Dysregulation	Samples (HCC/Control)	Description	Area under the ROC curve	Reference
*HULC*	Up	30/20	Increase with Edmondson grade, detected more frequently in HBV+ HCC patients	Not reported	[35]
	Up	90/77	Correlated with tumor size and tumor capsule	0.78	[36]

*Linc00152*	Up	90/77	Related to differentiation grade, tumor size, TNM stage and tumor capsule	0.85	[36]

*MALAT1*	Up	88/79	Significantly lower in HCC patients with hepatitis B infection and significantly higher in patients with liver damage B or liver cirrhosis	0.66	[41]

*HEIH*	Up	179/179	Positively related to TNM stage and the risk of HCC	0.681	[39]

*PVT1*	Up	71/64	Associated with clinical parameters including tumor size, BCLC stage and serum bilirubin	0.764 (combined with uc002mbe.2)	[43]

*uc002mbe.2*	Up	71/64	Same with PVT1	0.764 (combined with PVT1)	[43]

*UCA1*	Up	82/78	Associated with HCV-antibodies positive patients and Child-Pugh score	0.861	[40]

*WRAP53*	Up	82/78	Same with UCA1	0.896	[40]

*uc003wbd*	Up	137/138	Also significantly highly expressed in HBV patients	0.86	[37]

*AF085935*	Up	137/138	Same with uc003wbd	0.96	[37]

*uc001ncr*	Up	232/452	High diagnostic performance in patients with AFP <400 ng/ml and early HCC	0.8859	[42]

*AX800134*	Up	232/452	Same with uc001ncr	0.9251	[42]

*RP11-160H22.5*	Up	217/250	Decrease after operation	0.601	[38]

*XLOC_014172*	Up	217/250	Higher expression in HCC patients with metastasis	0.866	[38]

*LOC149086*	Up	217/250	Same with XLOC_014172	0.759	[38]

## References

[1] Venook A. P., Papandreou C., Furuse J., de Guevara L. L. (2010). The incidence and epidemiology of hepatocellular carcinoma: a global and regional perspective. *The Oncologist*.

[2] Villanueva A., Minguez B., Forner A., Reig M., Llovet J. M. (2010). Hepatocellular carcinoma: novel molecular approaches for diagnosis, prognosis, and therapy. *Annual Review of Medicine*.

[3] Kataoka M., Wang D. (2014). Non-coding RNAs including miRNAs and lncRNAs in cardiovascular biology and disease. *Cells*.

[4] Wang Y., Gao S., Liu G., Jia R., Fan D., Feng X. (2014). Microarray expression profile analysis of long non-coding RNAs in human gastric cardiac adenocarcinoma. *Cellular Physiology and Biochemistry*.

[5] Liu H., Song G., Zhou L. (2013). Compared analysis of LncRNA expression profiling in pdk1 gene knockout mice at two time points. *Cellular Physiology and Biochemistry*.

[6] Qiu M.-T., Hu J.-W., Yin R., Xu L. (2013). Long noncoding RNA: an emerging paradigm of cancer research. *Tumor Biology*.

[7] Li C. H., Chen Y. (2013). Targeting long non-coding RNAs in cancers: progress and prospects. *The International Journal of Biochemistry & Cell Biology*.

[8] Li C., Chen J., Zhang K., Feng B., Wang R., Chen L. (2015). Progress and prospects of long noncoding RNAs (lncRNAs) in hepatocellular carcinoma. *Cellular Physiology and Biochemistry*.

[9] Panzitt K., Tschernatsch M. M. O., Guelly C. (2007). Characterization of HULC, a novel gene with striking up-regulation in hepatocellular carcinoma, as noncoding RNA. *Gastroenterology*.

[10] Wang J., Liu X., Wu H. (2010). CREB up-regulates long non-coding RNA, HULC expression through interaction with microRNA-372 in liver cancer. *Nucleic Acids Research*.

[11] Du Y., Kong G., You X. (2012). Elevation of highly up-regulated in liver cancer (HULC) by hepatitis B virus X protein promotes hepatoma cell proliferation via down-regulating p18. *Journal of Biological Chemistry*.

[12] Cui M., Xiao Z., Wang Y. (2015). Long noncoding RNA HULC modulates abnormal lipid metabolism in hepatoma cells through an miR-9-mediated RXRA signaling pathway. *Cancer Research*.

[13] Cui M., Zheng M., Sun B., Wang Y., Ye L., Zhang X. (2015). A Long Noncoding RNA Perturbs the Circadian Rhythm of Hepatoma Cells to Facilitate Hepatocarcinogenesis. *Neoplasia*.

[14] Lu Z., Xiao Z., Liu F. (2016). Long non-coding RNA HULC promotes tumor angiogenesis in liver cancer by up-regulating sphingosine kinase 1 (SPHK1). *Oncotarget*.

[15] Li S., Xu H., Yu Y. (2016). LncRNA HULC enhances epithelial-mesenchymal transition to promote tumorigenesis and metastasis of hepatocellular carcinoma via the miR-200a-3p/ZEB1 signaling pathway. *Oncotarget*.

[16] Lin R., Maeda S., Liu C., Karin M., Edgington T. S. (2007). A large noncoding RNA is a marker for murine hepatocellular carcinomas and a spectrum of human carcinomas. *Oncogene*.

[17] Lai M.-C., Yang Z., Zhou L. (2012). Long non-coding RNA MALAT-1 overexpression predicts tumor recurrence of hepatocellular carcinoma after liver transplantation. *Medical Oncology*.

[18] Luo F., Sun B., Li H. (2016). A MALAT1/HIF-2*α* feedback loop contributes to arsenite carcinogenesis. *Oncotarget*.

[19] Wu Y., Liu X., Zhou Q. (2015). Silent information regulator 1 (SIRT1) ameliorates liver fibrosis via promoting activated stellate cell apoptosis and reversion. *Toxicology and Applied Pharmacology*.

[20] Rinn J. L., Kertesz M., Wang J. K. (2007). Functional demarcation of active and silent chromatin domains in human HOX loci by noncoding RNAs. *Cell*.

[21] Schorderet P., Duboule D. (2011). Structural and functional differences in the long non-coding RNA hotair in mouse and human. *PLoS Genetics*.

[22] Yang Z., Zhou L., Wu L.-M. (2011). Overexpression of long non-coding RNA HOTAIR predicts tumor recurrence in hepatocellular carcinoma patients following liver transplantation. *Annals of Surgical Oncology*.

[23] Geng Y. J., Xie S. L., Li Q., Ma J., Wang G. Y. (2011). Large intervening non-coding RNA HOTAIR is associated with hepatocellular carcinoma progression. *The Journal of International Medical Research*.

[24] Ishibashi M., Kogo R., Shibata K. (2013). Clinical significance of the expression of long non-coding RNA HOTAIR in primary hepatocellular carcinoma. *Oncology Reports*.

[25] Ding C., Cheng S., Yang Z. (2014). Long non-coding RNA HOTAIR promotes cell migration and invasion via down-regulation of RNA binding motif protein 38 in hepatocellular carcinoma cells. *International Journal of Molecular Sciences*.

[26] Fu W.-M., Zhu X., Wang W.-M. (2015). Hotair mediates hepatocarcinogenesis through suppressing miRNA-218 expression and activating P14 and P16 signaling. *Journal of Hepatology*.

[27] Gao J.-Z., Li J., Du J.-L., Li X.-L. (2016). Long non-coding RNA HOTAIR is a marker for hepatocellular carcinoma progression and tumor recurrence. *Oncology Letters*.

[28] Yang L., Zhang X., Li H., Liu J. (2016). The long noncoding RNA HOTAIR activates autophagy by upregulating ATG3 and ATG7 in hepatocellular carcinoma. *Molecular BioSystems*.

[29] Yuan S.-X., Yang F., Yang Y. (2012). Long noncoding RNA associated with microvascular invasion in hepatocellular carcinoma promotes angiogenesis and serves as a predictor for hepatocellular carcinoma patients' poor recurrence-free survival after hepatectomy. *Hepatology*.

[30] Shi Y., Song Q., Yu S., Hu D., Zhuang X. (2015). Microvascular invasion in hepatocellular carcinoma overexpression promotes cell proliferation and inhibits cell apoptosis of hepatocellular carcinoma via inhibiting miR-199a expression. *OncoTargets and Therapy*.

[31] Barsotti A. M., Beckerman R., Laptenko O., Huppi K., Caplen N. J., Prives C. (2012). p53-dependent induction of PVT1 and miR-1204. *The Journal of Biological Chemistry*.

[32] Wang F., Yuan J.-H., Wang S.-B. (2014). Oncofetal long noncoding RNA PVT1 promotes proliferation and stem cell-like property of hepatocellular carcinoma cells by stabilizing NOP2. *Hepatology*.

[33] Ding C., Yang Z., Lv Z. (2015). Long non-coding RNA PVT1 is associated with tumor progression and predicts recurrence in hepatocellular carcinoma patients. *Oncology Letters*.

[34] Chauhan R., Lahiri N. (2016). Tissue- and serum-associated biomarkers of hepatocellular carcinoma. *Biomarkers in Cancer*.

[35] Xie H., Ma H., Zhou D. (2013). Plasma HULC as a promising novel biomarker for the detection of hepatocellular carcinoma. *BioMed Research International*.

[36] Li J., Wang X., Tang J. (2015). HULC and Linc00152 Act as novel biomarkers in predicting diagnosis of hepatocellular carcinoma. *Cellular Physiology and Biochemistry*.

[37] Lu J., Xie F., Geng L., Shen W., Sui C., Yang J. (2014). Investigation of serum lncRNA-uc003wbd and lncRNA-AF085935 expression profile in patients with hepatocellular carcinoma and HBV. *Tumor Biology*.

[38] Tang J., Jiang R., Deng L., Zhang X., Wang K., Sun B. (2015). Circulation long non-coding RNAs act as biomarkers for predicting tumorigenesis and metastasis in hepatocellular carcinoma. *Oncotarget*.

[39] Zhefeng Y., Juan L., Zhiyuan M., Xisheng Y., Bin D., Jiaze A. (2015). Association between expression of LncRNA-HEIH in plasma and hepatocellular carcinoma risk: a case-control study. *Modern Oncology*.

[40] Kamel M. M., Matboli M., Sallam M., Montasser I. F., Saad A. S., El-Tawdi A. H. F. (2016). Investigation of long noncoding RNAs expression profile as potential serum biomarkers in patients with hepatocellular carcinoma. *Translational Research*.

[41] Konishi H., Ichikawa D., Yamamoto Y. (2016). Plasma level of metastasis-associated lung adenocarcinoma transcript 1 is associated with liver damage and predicts development of hepatocellular carcinoma. *Cancer Science*.

[42] Wang K., Guo W. X., Li N. (2015). Serum LncRNAs profiles serve as novel potential biomarkers for the diagnosis of HBV-positive hepatocellular carcinoma. *PLoS ONE*.

[43] Yu J., Han J., Zhang J. (2016). The long noncoding RNAs PVT1 and uc002mbe.2 in sera provide a new supplementary method for hepatocellular carcinoma diagnosis. *Medicine*.

[44] He Y., Lin J., Kong D. (2015). Current state of circulating microRNAs as cancer biomarkers. *Clinical Chemistry*.

[45] Hämmerle M., Gutschner T., Uckelmann H. (2013). Posttranscriptional destabilization of the liver-specific long noncoding RNA HULC by the IGF2 mRNA-binding protein 1 (IGF2BP1). *Hepatology*.

[46] Tripathi V., Ellis J. D., Shen Z. (2010). The nuclear-retained noncoding RNA MALAT1 regulates alternative splicing by modulating SR splicing factor phosphorylation. *Molecular Cell*.

[47] Quagliata L., Matter M. S., Piscuoglio S. (2014). Long noncoding RNA HOTTIP/HOXA13 expression is associated with disease progression and predicts outcome in hepatocellular carcinoma patients. *Hepatology*.

[48] Tsang F. H. C., Au S. L. K., Wei L. (2015). Long non-coding RNA HOTTIP is frequently up-regulated in hepatocellular carcinoma and is targeted by tumour suppressive miR-125b. *Liver International*.

[49] Ge Y., Yan X., Jin Y. (2015). fMiRNA-192 and miRNA-204 directly suppress lncRNA HOTTIP and interrupt GLS1-mediated glutaminolysis in hepatocellular carcinoma. *PLoS Genetics*.

[50] Yang F., Zhang L., Huo X.-S. (2011). Long noncoding RNA high expression in hepatocellular carcinoma facilitates tumor growth through enhancer of zeste homolog 2 in humans. *Hepatology*.

[51] Deng L., Yang S.-B., Xu F.-F., Zhang J.-H. (2015). Long noncoding RNA CCAT1 promotes hepatocellular carcinoma progression by functioning as let-7 sponge. *Journal of Experimental and Clinical Cancer Research*.

[52] Xu W. H., Zhang J. B., Dang Z. (2014). Long non-coding RNA URHC regulates cell proliferation and apoptosis via ZAK through the ERK/MAPK signaling pathway in hepatocellular carcinoma. *International Journal of Biological Sciences*.

[53] Sui C.-J., Zhou Y.-M., Shen W.-F. (2016). Long noncoding RNA GIHCG promotes hepatocellular carcinoma progression through epigenetically regulating miR-200b/a/429. *Journal of Molecular Medicine*.

[54] Tang J., Zhuo H., Zhang X. (2014). A novel biomarker Linc00974 interacting with KRT19 promotes proliferation and metastasis in hepatocellular carcinoma. *Cell Death and Disease*.

[55] Barsyte-Lovejoy D., Lau S. K., Boutros P. C. (2006). The c-Myc oncogene directly induces the H19 noncoding RNA by allele-specific binding to potentiate tumorigenesis. *Cancer Research*.

[56] Tsang W. P., Kwok T. T. (2007). Riboregulator H19 induction of MDR1-associated drug resistance in human hepatocellular carcinoma cells. *Oncogene*.

[57] Lv J., Yu Y.-Q., Li S.-Q., Luo L., Wang Q. (2014). Alatoxin B1 promotes cell growth and invasion in hepatocellular carcinoma HepG2 cells through H19 and E2F1. *Asian Pacific Journal of Cancer Prevention*.

[58] Zhang L., Yang F., Yuan J.-H. (2013). Epigenetic activation of the MiR-200 family contributes to H19-mediated metastasis suppression in hepatocellular carcinoma. *Carcinogenesis*.

[59] Lv J., Ma L., Chen X.-L., Huang X.-H., Wang Q. (2014). Downregulation of LncRNAH19 and MiR-675 promotes migration and invasion of human hepatocellular carcinoma cells through AKT/GSK-3*β*/Cdc25A signaling pathway. *Journal of Huazhong University of Science and Technology - Medical Science*.

[60] Zhou Y., Zhong Y., Wang Y. (2007). Activation of p53 by MEG3 non-coding RNA. *The Journal of Biological Chemistry*.

[61] Benetatos L., Vartholomatos G., Hatzimichael E. (2011). MEG3 imprinted gene contribution in tumorigenesis. *International Journal of Cancer*.

[62] Braconi C., Kogure T., Valeri N. (2011). MicroRNA-29 can regulate expression of the long non-coding RNA gene MEG3 in hepatocellular cancer. *Oncogene*.

[63] Zhou Y., Zhang X., Klibanski A. (2012). MEG3 noncoding RNA: a tumor suppressor. *Journal of Molecular Endocrinology*.

[64] Zhuo H., Tang J., Lin Z. (2016). The aberrant expression of MEG3 regulated by UHRF1 predicts the prognosis of hepatocellular carcinoma. *Molecular Carcinogenesis*.

[65] Zhu J., Liu S., Ye F. (2015). Long noncoding RNA MEG3 interacts with p53 protein and regulates partial p53 target genes in hepatoma cells. *PLoS ONE*.

[66] Chang L., Wang G., Jia T. (2016). Armored long non-coding RNA MEG3 targeting EGFR based on recombinant MS2 bacteriophage virus-like particles against hepatocellular carcinoma. *Oncotarget*.

[67] Huang J. F., Guo Y. J., Zhao C. X. (2013). Hepatitis B virus X protein (HBx)-related long noncoding RNA (lncRNA) down-regulated expression by HBx (Dreh) inhibits hepatocellular carcinoma metastasis by targeting the intermediate filament protein vimentin. *Hepatology*.

[68] Yang F., Huo X.-S., Yuan S.-X. (2013). Repression of the long noncoding RNA-LET by histone deacetylase 3 contributes to hypoxia-mediated metastasis. *Molecular Cell*.

